# Designing grazing susceptibility to land degradation index (GSLDI) in hilly areas

**DOI:** 10.1038/s41598-022-13596-1

**Published:** 2022-06-21

**Authors:** Gabriel Minea, Nicu Ciobotaru, Gabriela Ioana-Toroimac, Oana Mititelu-Ionuș, Gianina Neculau, Yeboah Gyasi-Agyei, Jesús Rodrigo-Comino

**Affiliations:** 1 Research Institute of the University of Bucharest, 90 Sos. Panduri, 5th Sector, 050663 Bucharest, Romania; 2 National Institute of Hydrology and Water Management, 97E București-Ploiești Road, 1st Sector, 013686 Bucharest, Romania; 3 Faculty of Geography, University of Bucharest, 1 Nicolae Bălcescu, 1st Sector, 010041 Bucharest, Romania; 4 Department of Geography, Faculty of Sciences, University of Craiova, 13 A.I. Cuza Street, 200585 Craiova, Romania; 5 School of Engineering and Built Environment, Griffith University, Nathan, QLD 4111 Australia; 6 Departamento de Análisis Geográfico Regional y Geografía Física, Facultad de Filosofía y Letras, Campus Universitario de Cartuja, University of Granada, 18071 Granada, Spain

**Keywords:** Environmental impact, Geomorphology, Hydrology, Environmental sciences

## Abstract

Evaluation of grazing impacts on land degradation processes is a difficult task due to the heterogeneity and complex interacting factors involved. In this paper, we designed a new methodology based on a predictive index of grazing susceptibility to land degradation index (GSLDI) built on artificial intelligence to assess land degradation susceptibility in areas affected by small ruminants (SRs) of sheep and goats grazing. The data for model training, validation, and testing consisted of sampling points (erosion and no-erosion) taken from aerial imagery. Seventeen environmental factors (e.g., derivatives of the digital elevation model, small ruminants’ stock), and 55 subsequent attributes (e.g., classes/features) were assigned to each sampling point. The impact of SRs stock density on the land degradation process has been evaluated and estimated with two extreme SRs’ density scenarios: absence (no stock), and double density (overstocking). We applied the GSLDI methodology to the Curvature Subcarpathians, a region that experiences the highest erosion rates in Romania, and found that SRs grazing is not the major contributor to land degradation, accounting for only 4.6%. This methodology could be replicated in other steep slope grazing areas as a tool to assess and predict susceptible to land degradation, and to establish common strategies for sustainable land-use practices.

## Introduction

Unsustainable grazing is a millennial activity that can trigger land degradation processes if appropriate soil management practice is not put in place^1–3^. However, it is a vital activity that ensures food security^4^ and ecosystem health^5^.

Pastoral land activities are some of the most common farming practices in many parts of the world such as central and southeastern Europe. Some representative areas are located in Poland and Romania^6,7^ and along the Pyrenees in Spain^8^. Livestock is in many countries the largest agricultural sector and occupies about 37% of the global ice-free land surface (130.4 M km^2^)^9^. Across the world, the domestic stock was estimated as 1,238 billion sheep and 1094 billion goats in 2019^10^, which delivered 1/3 of all protein consumed by humanity^11^. Small ruminants (SRs) stock (sheep and goats) can be considered a major contributor to the global economy accounting for about 2% (1995–2005) of the global gross domestic product^12,13^. In the context of increasing demand for food due to the growing human population, the environmental impact of various sectors of agriculture will also increase^14^. The increasing combined pressure of agricultural and livestock productions (e.g., unsustainable grazing) is assumed as one of the main factors expected to accelerate land degradation in the twentieth and twenty first centuries^15^. Soil erosion in agricultural areas^16–19^ and intensive grazing are recognized as global environmental issues^20–22^.

Grazing, as a component of land use, is associated with certain patterns in hydrosedimentary conditions from event-scale to the catchment scale (e.g.,^23,24^ and several studies have analyzed the relationship between unsustainable grazing, grassland status, and animal types (e.g.,^25–32^. Unsustainable and prolonged grazing may trigger hydrological changes such as soil water content, but also the activation of runoff with an impact on the streamflow regime (e.g., maximum and minimum flow) affecting runoff coefficients^33–37^, even with modifications of the terrestrial water^38^. For example, Meyles et al.^39^ assessed flood frequency with a higher occurrence of erosion in large areas under intensive grazing process in a small Dartmoor catchment, in southwest England. Poesen^40^ stressed the need for more research attention on the hydrological and erosional response of rangelands. Hancock et al.^41^ quantified erosion in a grazing environment typical of the east coast of Australia and discovery that under current management practices soil loss is relatively low (< 5 t ha^−1^ y^−1^).

Most hydrological studies have underlined the role that vegetation plays in grazing lands in controlling erosion^42^ and the biocrust^43,44^. Some authors pointed out that about 20% of the world’s pasture areas are considered degraded as a consequence of overgrazing and associated erosion and compaction among other main factors^13^. Nowadays, scholars have highlighted that this relationship is complicated and not well understood. One of the most complex mechanisms is related to the SRs where they can influence organic matter and facilitate the adherence of aggregated clay and the formed colluvial layer that serves as a substrate to the expanded vegetation growth^45,46^. Moreover, satellite-derived data such as normalized difference vegetation index (NDVI) can be used in land degradation assessments, but without distinguishing specific signs of degradation/conservation from impacts of adverse/beneficial natural processes^5,47^. In the last few years, in the temperate climate of Europe, the analysis of SRs grazing influence on soil and runoff processes has received minimal interest but a few devoted field works have been carried out at the hillslope scales under grazing (e.g.,^28,48–51^.

Some previous methods and tools used for the quantification of grazing as part of land degradation and erosion have shown several drawbacks. For example, field-based monitoring has to be planned for very long-term periods to foresee future changes, which may become expensive and time-consuming^52^. Therefore, the lack of monitoring and observational data is hampering the assessment of land degradation by grazing. Nowadays, remote sensing techniques and modeling approaches based on landscape unit and vegetation indexes are becoming useful tools. Riva et al.^47^ showed that grazing is a significant cause of land degradation even in partially abandoned areas, and the effectiveness of responses to land degradation is substantially affected by land cover and topography.

Given the spatial and temporal variability of various grazing practices that complicates the interpretation within mechanistic models, Ma et al.^53^ highlighted that efforts are needed to reduce inconsistencies among grazing land models in simulated grazing management effects by carefully examining the underlying processes interacting in each model. Also, interesting results were obtained by Kosmas et al.^54^ using geographical information systems (GIS) at the local scale, concluding that rapid growth in the livestock density increased soil erosion rates. Currently, management of natural environmental hazards (e.g., floods, landslides, gully can be assessed, mapped and predicted using machine learning (ML, a branch of artificial intelligence, algorithms and tools. More devoted literature using ML combined with diverse statistical methods (e.g., random forest—RF; boosted regression tree—BRT can be found in valuable scientific works focused on vulnerable territories (e.g., Himalayan regions, for example by Roy et al.^55^, Chowdhuri et al.^56,57^ Pal et al.^58^, Costache et al.^59,60^ and Vojtek et al.^61^.

Thus, the relationship between land degradation and unsustainable grazing in Europe is still poorly understood and should be further assessed^40,51,62,63^. Degraded land occupies over 2% of Romania due to corroboration of natural and anthropogenic causes^64^. Recent studies confirmed land degradation by soil erosion^65^, gullying and landslides^66^, and reservoir sedimentation^67^, and the statistics of stocking rate and grazing deviate from the optimum^68^. For the Southern Carpathian Mts., Romanian, transhumance of sheep prevents the development of serious erosion in otherwise erosion-prone areas that can support little beyond livestock raising^69^. Surprisingly, Nicu^70^ found that overgrazing is not influencing or accelerating soil erosion on gullies in the Bahluiet River catchment in the northeastern part of Romania. In this context, land degradation under grazing activities is a serious problem in hilly areas of Romania and effective soil conservation is urgently needed. Yet, it remains a knowledge gap in the quantification of the role of grazing among numerous factors on erosion and land degradation in past interdisciplinary investigations at the regional scale. Filling the gap of quantifying the complexity of land degradation drivers could help decision-makers in spatial planning and agriculture to set guidelines for land-use policy.

Therefore, this paper starts from the hypothesis that land degradation and grazing can be assessed using multiple environmental factors, and even at larger scales. The main aim of this research is to quantify the relation between grazing impacts of SRs on land degradation processes using the Grazing Susceptibility to Land Degradation Index (GSLDI). The specific objectives are:

1. To develop a novel model using a qualitative assessment of the evidence-support of a hypothesis that integrates weight/partitive factors of land degradation at a regional scale; and
2. To investigate a hilly region in Romania (the Curvature Subcarpathians) that is characterized by the highest rates of erosion and SRs density with GSLDI.

### Geographic background of the surveyed area

We adopted for the survey of GSLDI a geomorphological unit for 3 reasons; relative geomorphological homogeneity^71^, being one of the largest areas with historical land degradation (badlands) in Romania^72,73^, and having the highest value of sediment yield^74–76^. However, this research starts from the premise that land degradation processes are also due to other local and regional factors (e.g., lithology, morphology, land use). Land-use changes should be considered, as well as agricultural practices such as grazing. Therefore, it was necessary to apply an integrated analysis of the possible determinant factors’ assembly to understand and manage the land degradation in the Curvature Subcarpathians.

**Figure 1:**
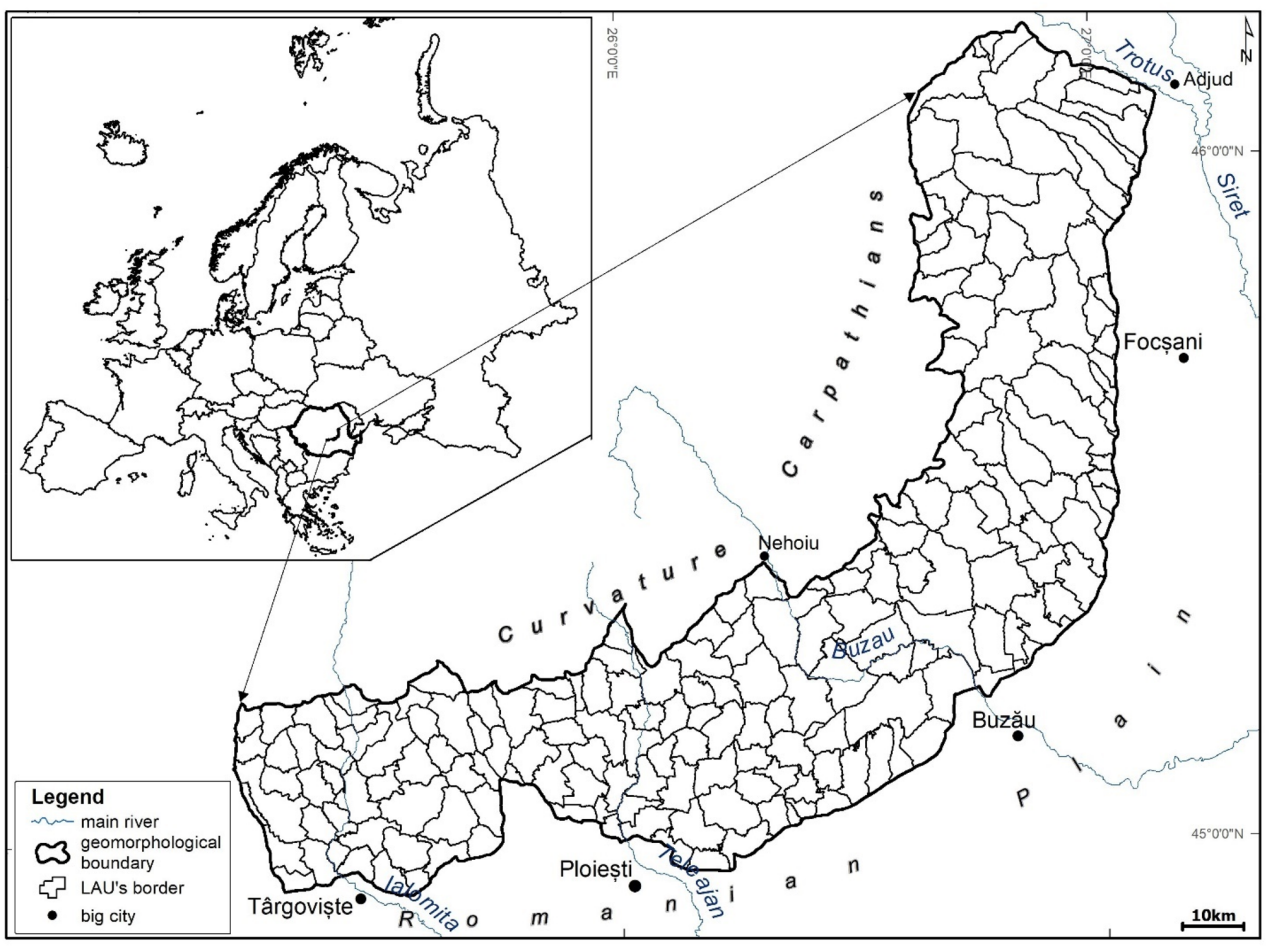
The location of Curvature Subcarpathians and the border of Local Administrative Units level 2. The geographic data vector comes from the https://geo-spatial.org; map created by G. Minea using ArcGIS Pro version 2.8.6, https://pro.arcgis.com/en/pro-app/2.8/get-started/get-started.htm.

The Curvature Subcarpathians (6792 km sq km) is a geomorphologic region in the central part of Romania that lies between latitudes 44°47′01.77″ to 46°09′58.43″N and longitudes 24°11′13.00″ to 27°59′34.82″E (Stereographic 1970 projection), having a southern border with the Curvature Carpathians Mts (Fig. 1). The study area is prone to land degradation, mainly erosion, due to both natural and socio-economic drivers (e.g., historical deforestation). The estimated peak erosion in Romania occurs in the Curvature Subcarpathians with rates between 30 and 45 t ha^−1^ y^−1^^75,76^. Besides denudation, the Curvature Subcarpathians are characterized by various types of slope processes such as landslides, earth flows, and falls^77^, and also by an intense vertical erosion mirrored by incised river valleys (Buzău, Ialomiţa and Teleajen Rivers)^78^. The lithology and seismicity explain the susceptibility to landslides^79^, the predominance of low cohesive rocks, sandy clay loam and clay soils, and active neotectonic movements^80^,^73,77^. Persistent deforestation may also trigger gravitational processes. The natural vegetation (e.g., broad-leaved forests) has been replaced by human activities with secondary grasses, orchards, vineyards, and croplands. The road network has amplified the disequilibrium of slopes.

The Curvature Subcarpathians receive approximately 600–700 mm of precipitation per year, the maximum occurring in summer (up to 100 mm/month), and the lowest in autumn and winter^81^. Summer months are characterized by heavy rains that transform into overland flow causing aggressive erosion, especially on the bare soil^78^. The study area appears to be among the regions with the national highest maximum rainfall intensity of 5 min duration and 1:10 years return period (e.g., 1.48–1.86 mm/min reported by^82^.

Rivers draining the Curvature Subcarpathians transport large amounts of suspended sediment load^83^. As a consequence, the largest rivers form sandy braided patterns, laterally unstable^84^. Also, the study area is characterized by the highest suspended sediment yield in Romania^74,81^. The mean specific suspended sediment yield is about 20–25 t ha^−1^ yr^−1^, while the sediment concentration exceeds 25,000 g m^−3^^85^. The highest suspended sediment load occurs in spring and summer during the high-water phase of the hydrological regime, or during floods, when the fluvial erosion, or erosion processes, on the slopes are more intense. It is observed that the variability of the suspended sediment load is higher than the variability of the mean annual water discharge^86^.

## Methods and data

### Methodological steps

Based on the recent approach by Costacheetal.^59,60^ and Vojtek et al.^61^, we developed a new advanced GSLDI and the SRs grazing pressure using ML algorithms. The proposed modeling framework consists of: (1) land erosion inventory (manually identifying erosion and no-erosion sampling points based on aerial imagery); (2) developing geographical characteristics (17 parameters, e.g., lithological structure, altitude) for each sampling point, (3) standardizing the dataset (55 subsequent attributes e.g., classes/features), and (4) ML model training process.

### Data processing

The database for land erosion inventory requires compulsory sampling points (vector data) exhibiting erosion or no-erosion processes detected using open-source imagery (e.g., Google Earth, Bing) for the study area. Manual collection of sampling points data time consuming and should be harmonized (checks, validation) with the laboratory staff. Moreover, the density of the sampling points can be varied between low (e.g., forest sites) and high (land-use fragmentation) and should be agreed upon in accordance with the land-use characteristics.

**Figure 2:**
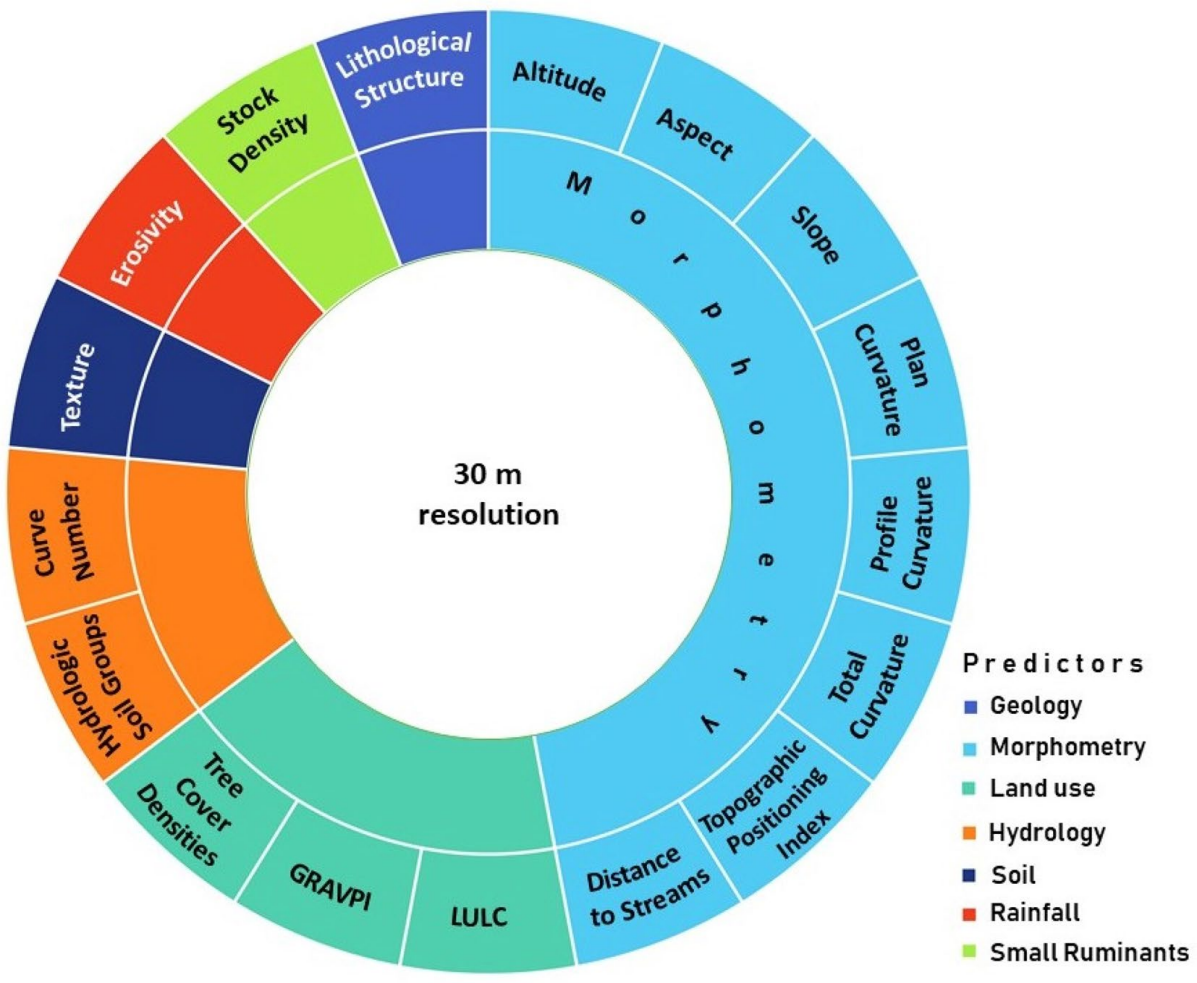
The proposed hierarchy predictors and their geographical use to develop the Grazing Susceptibility to Land Degradation Index. (GRAVIPA = Grassland Vegetation Probability Index; LULC = Land Use Land Cover). The figure was shaped by G. Minea using Microsoft 365 PowerPoint (https://www.microsoft.com/en-us/microsoft-365/powerpoint).

A database consisting of 17 geographical parameters (e.g., curvature, slope terrain) converted into thematic layers was extracted from a digital elevation model (DEM); lithology and soil maps^87^; vegetation features (e.g., Corine Land Cover); rainfall erosivity^88^, and SRs parameters (stock and densities), as presented in Fig. 2. These datasets were used to train and validate the proposed model. In accordance with Brock et al.^89^, we used in this study the Shuttle Radar Topography Mission (SRTM) 1 Arc-Second Global DEM product. Available vector (e.g., boundary shapefile for the geomorphologic region and the Local Administrative Units—LAU) and raster format data (e.g., at medium-precision, the scale of 1:25,000) and large maps (e.g., lithological and pedological maps 1:200,000) were required in the database. We chose a regional geographical approach combined with the stock density data of SRs mainly because SRs rank as land degradation significant variables.

To implement this analysis, several spreadsheet calculations were required, followed by geospatial processing (e.g., vector to raster format; union/merge, reclassify). A horizontal spatial grid resolution of 30 m × 30 m was used, and also resamples were necessary (Fig. 2).

The model training procedure involved evaluation of the two ML classification models of random forest (RF) and gradient boosted machine (GBM) using some performance statistics.

**Figure 3:**
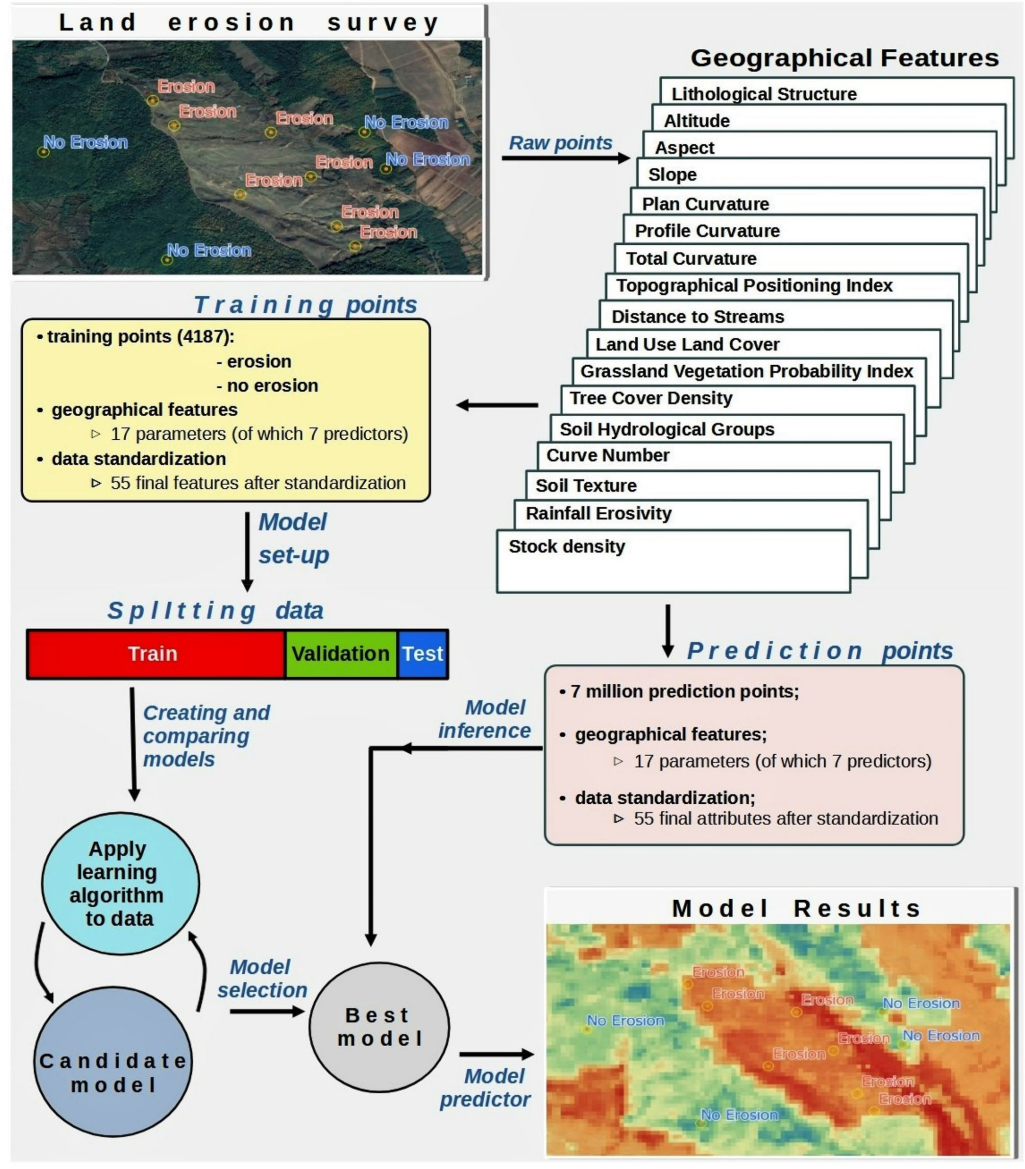
Workflow for designing Grazing Susceptibility to Land Degradation Index. The figure was designed by N. Ciobotaru in Microsoft 365 PowerPoint (https://www.microsoft.com/en-us/microsoft-365/powerpoint).

The GIS environment of the modeling process requires an association of the 17 geographical features (e.g., lithological structure, altitude) with subsequent attributes (e.g., classes/features) for each sampling point (Figs. 2, 3). The classes of the geographical features (e.g., elevation range) were determined based on a statistical approach (e.g., Natural breaks classification).

To calculate the GSLDI, the ML models were trained to predict erosion exposure by using the 17 attributes and their classes as predictors, resulting in the probability of having erosion process on each pixel of the study area. The model set-up consists of randomly splitting the data (erosion and no-erosion points) into two categories: training dataset (80%) and the test dataset. The test dataset was used to evaluate the model performance independently as it was not part of the training phase (Fig. 3). The validation set was drawn from the training dataset by means of tenfold cross-validation procedure.

To evaluate and estimate the impact of SRs stock density over the land degradation process, we propose two SRs’ density scenarios: one considered 0 (absence), and another one with double density (overstocking).

The outcomes were presented in a probabilistic map of a chance between 0–100 per cent to have exposure to the land degradation process concerning SRs, that resulted in a map of land degradation (Fig. 3). The results of the analysis were plotted in a GIS environment, using the QGIS version 3.20 and ArcGIS Pro version 2.8.6 software, and R programming language with RF, caret, GBM, and other auxiliary packages.

### The classification algorithms

Numerous studies have used random forest (RF) models to evaluate erosion process (e.g.,^90–92^, gully erosion susceptibility (e.g.,^56–58,93^, floods mapping (e.g.,^55,94^) and groundwater nitrate concentration susceptibility^95^. Random decision forests were first introduced by Ho^96^ who added randomness in the decision trees with increased accuracy for both training and unseen datasets. This method builds multiple trees in “*randomly selected subspaces of the feature space*” and generates the final output by averaging results from all created decision trees. In this way, bias is reduced and accuracy improves. RF is a method of ensemble learning by combining multiple decision trees over the same classification task. The output of the RF algorithm is the class that is selected by most trees in the forest.

Given *t* trees created in random subspaces, a discriminant function is used to combine the classification result by assigning *x* to class *c* and optimising the function $g_{c} \left( x \right)$ defined as^96^:

1 $$g_{c} \left( x \right) = \frac{1}{t}\mathop \sum \limits_{j = 1}^{t} \hat{P}\left( {c|v_{j} \left( x \right)} \right)$$

In Eq. (1) $\hat{P}$ is the optimum posterior probability that a point *x* belongs to class *c* (*c* = 1,2…n) and is computed as the fraction of class *c* that points to the overall number of points that are assigned to $v_{j} \left( x \right)$ given as:

2 $$P\left( {c|v_{j} \left( x \right)} \right) = \frac{{P\left( {c, v_{j} \left( x \right)} \right),}}{{\mathop \sum \nolimits_{i = 1}^{n} p\left( {c_{l} , v_{j} \left( x \right)} \right)}}$$

where $v_{j} \left( x \right)$ is the terminal node of point *x* when it descends down tree *T*_*j*_ (j = 1,2, …t).

Gradient boosted machine (GBM) has also been used in soil degradation/erosion susceptibility studies in different forms^97–99^ and was first introduced by Friedman et al.^100^. It is also an ensemble model of decision trees. Also, various innovative validation methods such as Nash–Sutcliffe Criteria^101^, Seed Cell Area Index^102–104^ and the K-fold cross-validation^95^ can be used to evaluate the performance of models. The difference between RF algorithms and GBM is that the combining process is done at the beginning of the tree in the case of GBM while for RF it is done at the end. Also, while RF builds each tree independently, GBM works by building one tree at a time, introducing a weak learner to improve the shortcomings of existing weak learners.

Let $\left\{ {\left( {x_{i} ,y_{i} } \right)} \right\}_{i = 1}^{n}$ be the training dataset and $L\left( {y, F\left( x \right)} \right)$ the loss function, where $\hat{y} = F\left( x \right)$ is the model predicted values, $y$ represents the observed values, *x* the predictors and n the number samples. First, the algorithm initializes with a constant value as:

3 $$F_{0} \left( x \right) = \arg min_{\rho } \mathop \sum \limits_{i = 1}^{N} L\left( {y_{i} ,\rho } \right)$$

Next, for $$m = 1\;{\text{to}}\;M$$:

1. Compute the *pseudo-residuals as:*
  4 $$y_{i} = - \left[ {\frac{{\partial L\left( {y_{i} ,F\left( {x_{i} } \right)} \right)}}{{\partial F\left( {x_{i} } \right)}}} \right]_{{F\left( x \right) = F_{m - 1} \left( x \right)}} ,\quad i = 1,N$$
2. Fit a weak learner by training it on the training set as:
  5 $$a_{m} = \arg min_{a,\beta } \mathop \sum \limits_{i = 1}^{N} \left[ {\widetilde{{y_{i} }} - \beta h\left( {x_{i} ;a} \right)} \right]^{2}$$
3. Solve optimization problems as:
  6 $$p_{m} = \arg min_{p} \mathop \sum \limits_{i = 1}^{N} L\left( {y_{i} ,F_{m - 1} \left( {x_{i} } \right) + ph\left( {x_{i} ;a_{m} } \right)} \right)$$
4. Update the model as:
  7 $$F_{m} \left( x \right) = F_{m - 1} \left( x \right) + p_{m} h\left( {x;a_{m} } \right)$$

Thus, a gradient boosting machine involves combining multiple weak classifiers in order to obtain a stronger one of the ensemble models.

### Model efficiency and accuracy evaluation

To train the ML models, it was necessary to standardize the different measurements and the qualitative attributes of points (e.g., soil texture) to the same scale with the help of the “*scale*” function of the “*dummies package*”^105^ of the R programming language. The cross-validation technique was used in the caret package in R^106^ to randomly select the training and validation datasets in 10 folds for both models.

The training was conducted using cross-validation, which randomly partitions the dataset into complementary subsets of training and testing to test the model’s ability to predict new data that was not used for training. Evaluation of model performance is a critical step toward the classification model selection criteria. The model results were evaluated using a confusion matrix and testing of the accuracy, specificity, sensitivity, ROC curve, and AUC curve efficiency metrics^102,106^. The criteria used to evaluate the models are presented below:

8 $$Sensitivity = \frac{TP}{{TP + FN}}$$

9 $$Specificity = \frac{TN}{{TN + FP}}$$

10 $$AUC = \frac{{\mathop \sum \nolimits_{n}^{i} TP + \mathop \sum \nolimits_{n}^{i} TN}}{{\left( {P + N} \right)}}$$

where *TP* = true positive, *TN* = true negative, *FP* = false positive, *FN* = false negative, *P* = all positives, and *N* = all negatives.

## Results and analysis

### Parameters selection and development

Firstly, we mapped 4187 sampling points on the Curvature Subcarpathians, indicating whether there is erosion or no-erosion processes, from Google, Esri, and Bing imagery that were available for August 2021, using the HCMGIS plugin from QGIS 3.20. Even though the images have been taken at different periods, using three sources allowed the observers to check whether a sampling point has been erroneously classified as an erosion or no-erosion point. within the end, 61% of the sampling points were classified as erosion points and the rest as no-erosion points.

**Figure 4:**
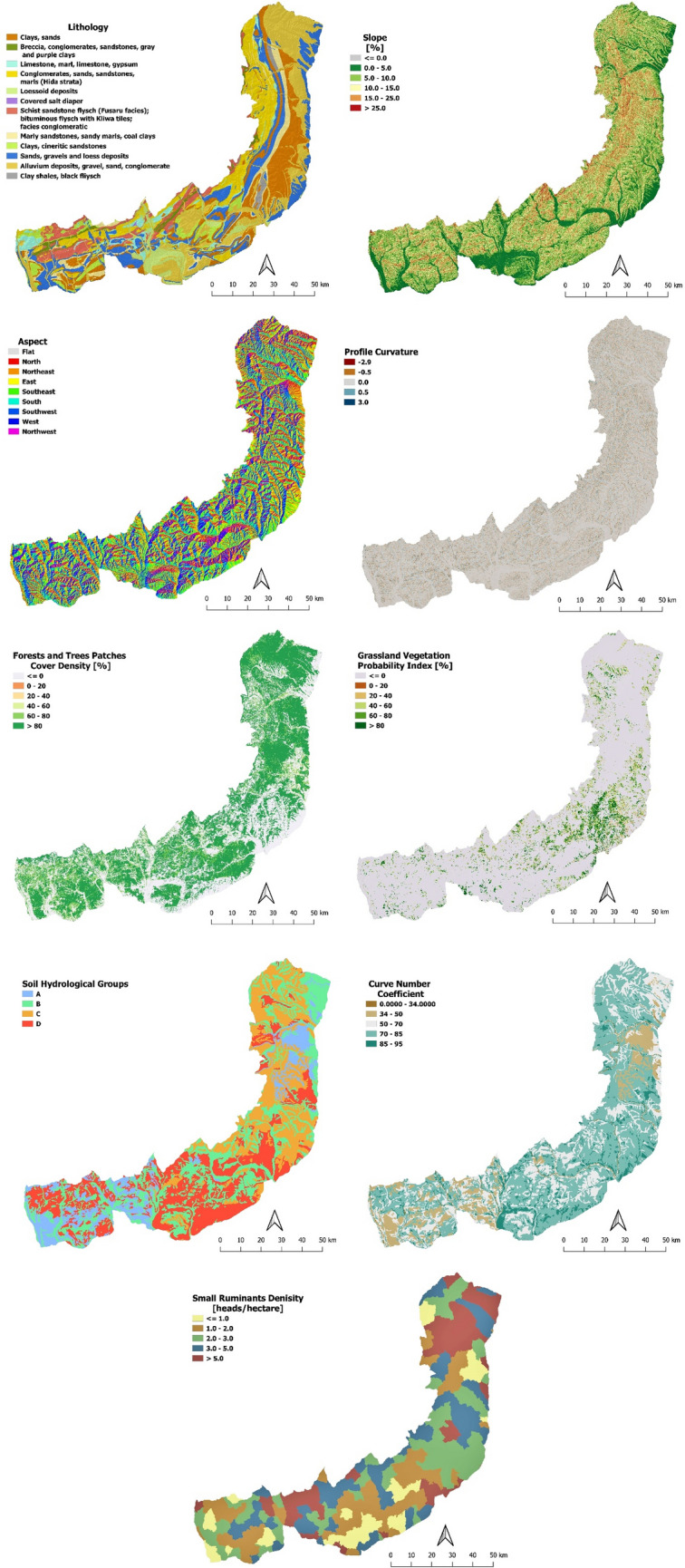
Curvature Subcarpathians geographic factors: (**a**) Lithology; (**b**) Slope angle; (**c**) Aspect; (**d**) Profile Curvature; (**e**) Forest and Trees Patches Cover Density; (**f**) GRAVPI; (**g**) Hydrologic Soil Groups; (**h**) Curve number; (**i**) SRs Density. The map was created by N. Ciobotaru using the QGIS version 3.20 (https://www.qgis.org).

From geo-spatial.org, we extracted datasets containing vector geospatial information such as LAUs Boundary dataset, localities dataset, and hydrography features dataset, and DEM of 30 m × 30 m spatial resolution as raster format. Some datasets were resampled to conform to the 30 m × 30 m grid resolution (e.g., Tree Cover Density). More relevant morphometric parameters/factors (e.g., slope; profile curvature) was derived from the DEM. Lithological and soil features were extracted from the Geological Map of Romania (scale of 1:200,000), and the Soil Map of Romania (scale of 1:200,000) respectively. LAUs (formerly NUTS level 5) was used as the smallest territorial subdivision by the Nomenclature of Territorial Units for Statistics (NUTS) for statistical purposes (see Supplementary 1). Therefore, each sampling point has 17 geographical attributes as presented in Table 1. In Fig. 4 is displayed maps of some predictors (such as lithology, soil properties, and land use land cover).

**Table 1:** Parameters used for training the sampling points.

Predictor	Parameter	Data source	Attributes (classes/features)	Area (km^2^)	Share of the total area (%)
Geology	Lithological structure	Geological Map of Romania (1:200,000)	Gravel	1608.8	23.6
			Clays	1201.3	17.6
			Sands	1161	17
			Conglomerates	903.5	13.3
			Flysch	438	6.4
			Sandstone	400.1	5.9
			Chalk–limestone	292.9	4.3
			Loess	296.4	4.3
			Marl	286.5	4.2
			Breccias	126.5	1.9
			Shale rock	92.9	1.4
			Diapir deposits	7.2	0.1
Morphometry	Altitude [m]	Digital Elevation Model^108^	0–300	1388.3	20.3
			300–500	3240.4	47.3
			500–800	2110.7	30.8
			800–1205	108.8	1.6
	Aspect		Flat	16.5	0.2
			North	623.8	9.1
			North-East	888.3	13
			East	1055.9	15.5
			South-East	1020.4	15
			South	924.6	13.6
			South West	901.9	13.2
			West	768.4	11.3
			North West	614	9
	Slope [%]		< 5	1473	21.6
			5–10	2268.7	33.3
			10–15	1986.6	29.1
			15–25	1038.7	15.3
			> 25	48.9	0.7
	Plan curvature		< − 0.5	74	1.1
			− 1	6646.1	97.5
			> 0.5	95.8	1.4
	Profile curvature		< − 0.5	68.6	1
			− 1	6670.4	97.9
			> 0.5	76.8	1.1
	Total curvature		< − 0.5	265.7	3.9
			− 1	6258.7	91.8
			> 0.5	291.4	4.3
	Topographic Positioning Index		Large valleys	756.1	11
			Hill top	967.2	14.1
			Mid slope	640.6	9.4
			Torrential valleys	889.2	13
			Plain or hil base	3594.8	52.5
	Distance to streams [m]		0–500	3971.9	59.4
			500–1000	2282.2	34.1
			1000–1500	421.1	6.3
			1500–2000	9.35	0.1
Soil	Texture	Soil Map of Romania (1:200,000)	Clay loam	5576.7	81.8
			Clay	1165.3	17.1
			Silty clay loam	42.9	0.6
			Loam	21	0.3
			Silty Clay	10	0.1
Land use	Tree cover density [%]	Copernicus Land Monitoring service; URL: https://land.copernicus.eu/pan-european/high-resolution-layers/forests/tree-cover-density/status-maps/tree-cover-density-2018	< 0.99	2499	36.7
			1–20	0.68	0.01
			21–40	8.05	0.1
			41–60	79.5	1.2
			61–80	993	14.6
			> 80	3232.7	47.4
	GRAVPI [%]	Copernicus Land Monitoring service; URL: https://land.copernicus.eu/looking-for-national-products	< 0.99	5913.2	86.8
			1–20	47	0.7
			21–40	111.8	1.6
			41–60	247.9	3.6
			61–80	258.3	3.8
			> 80	237.1	3.5
	LULC	Land Use Land Cover (2018); URL: https://land.copernicus.eu/pan-european/corine-land-cover/clc2018	Forest and trees	3212.6	47.1
			Pastures and natural grasslands	1400.4	20.5
			Agricultural land	1226.7	18
			Built	481.8	7.1
			Vineyards and trees	392.6	5.8
			Marshes and water courses	78.3	1.1
			Bare land	22.2	0.3
			Green urban areas	0.4	0.01
			Road and rail networks and associated land	0.3	0.01
Hydrological	Hydrologic soil groups	Derived from Soil Map of Romania (1:200,000)	A	963	14.1
			B	2166.8	31.8
			C	1657.1	24.3
			D	2028.7	29.8
	Curve number	Derived from Soil Map of Romania (1:200,000) and LULC (2018)	> 34	65.8	0.99
			34–50	753.6	11.3
			51–70	1848.7	27.8
			70–85	2767.9	41.6
			85–95	1214.4	18.3
Rainfall	Erosivity [(MJ mm)/(ha h yr)]	European Soil Data Center; URL: https://esdac.jrc.ec.europa.eu/resource-type/datasets	600–700	2.4	0.03
			700–800	1998.1	29.1
			800–900	4603.1	67.1
			900–1000	255.4	3.7
Small ruminants	Density [heads/ha]	General Agricultural Census^118^; URL: http://www.rga2010.djsct.ro/inceput.php?cod=58&codj=10) Land fund area by usage, counties and localities; URL: http://statistici.insse.ro:8077/tempo-online/#/pages/tables/insse-table)	< 1	771	11.3
			1–2	1503.1	22.1
			2–3	1710.7	25.1
			3–5	1576.3	23.1
			> 5	1254.6	18.4

*GRAVPI* grassland vegetation probability index, *LULC* land use land cover.

Some particularities of geographical attributes associated with erosion and no-erosion sampling points in the Curvature Subcarpathians are briefly debated. The lithological structure^107^ of the study region is dominated by Clays, Sands, and Conglomerates, indicating that the under-soil layer is unstable. Altitude, Slope, and Aspect were extracted from the Digital Elevation Model with the resolution of 30 m = ^108^. The classes and percentage shares of the total area are presented in Table 1. The altitudes are generally moderate (300–500 m), slopes are moderate (10–15%) to steep (15–25%) in 45% of the total area, while mild slopes (5–10%) dominate the landscape of the region, especially in the Plains and at the foothills. Aspect favors the exposure to the sun in general, the slopes being east, south-east, south, and southwest facing in over 57% of the area. Thus, the Curvature Subcarpathians region has a high insolation value which favors evapotranspiration, which in turn reduces water availability, and encourages the presence of thermophile vegetation such as vineyards, oak, Tilia forest, or common lilac.

Curvature is the second derivative of the land surface, indicating exposure to erosion of the landform area^109^. Profile curvature influences the acceleration/deceleration of flow while plan curvature indicates the concentration of flow on the land surface. Both profile and plan curvatures are indicators of the rate at which erosion and accumulation take place. These two characteristic values ranging between − 0.5 and 0.5 are not considered very active regarding the erosion process, while values above or below these thresholds represent areas most exposed to the erosion process. The total curvature combines these two indicators, and more than 8% of the territory is very exposed to the erosion process, compared with less than 3% when using profile and plan curvature.

Topographical positioning index (TPI) classifies the relief in different topographical units based on a change in altitude compared with the mean altitude of the transect with a radius of 1 km^110^. TPI describe the main landforms of the Subcarpathians region, resulting in over 52% of the relief occupied by flood plains combined with the base of the hills (basin) landforms, while the torrential valleys, most exposed to the erosion process, have a share of 13% of the total area.

Distance to Streams can be related to the erosive action of the river channels as the closer to the river channel the greater the chance to encounter land degradation processes. Therefore, this indicator allows us to create a relation between the observed soil degradation areas and the distance to the stream channel. This indicator has been extracted from a drainage network of support threshold of 1 km^2^ catchment area.

Corine LULC layer has been used in the study area as the main nomenclature for land use, and the spatial resolution of 1 ha was resampled to 30 m. It has been divided into 9 classes (label 3), of which 18% are covered by agricultural land, 7% are built areas and almost 6% are represented by vineyards and trees. The most important two classes are forests (47%) and pastures and grasslands (20%).

Grassland vegetation probability index (GRAVPI) constitutes an expert product estimation from the Copernicus Land Monitoring Service^111,112^ and is used to represent the spatial distribution of grassland areas within the study zone in a raster with the spatial resolution of 30 m. From this layer, it was observed that almost 87% of the area is covered with LULC types other than grassland areas. This is caused by the fact that the herbaceous zones are mixed with trees or bare land and agricultural land, making it difficult to identify this type of LULC, and in general, is underestimated compared with corine land cover (CLC) 2018.

Tree Cover Density from Copernicus Land Monitoring Service^113^ represents the density of trees (between 0 and 100%) in forested and non-forested areas in 2018, spatial resolution being 20 m. This dataset was resampled to 30 m to conform to the same resolution of the other layers used in the analysis.

The survey area has a forest cover of over 63%, of which 47% is in patches with densities above 80%, thus considered a compact forest. The overall cover of 63% is an overestimation of the forest cover, compared with the CLC layer, whereas only 47% of the study zone is classified as forest. This difference is explained by the fact that tree cover density is evaluated in all patches of trees, without considering the mixture of pastures, or agricultural areas, with the trees.

Hydrologic Soil Groups represent the rate at which water infiltrates into the soil, Soil Group A being the most porous (with over 7.6 mm/h infiltration rate) while Group D corresponds to soils with lower permeability (0–1.3 mm/h)^114^. This dataset has been derived from soil texture classes of the Romanian Soil dataset 1:200,000^115^, using the method developed by Drobot^116^. The areas with low permeability (Group D) are the most exposed to soil erosion, favoring surface and rill erosion in the Curvature Subcarpatians. These soils cover almost 30% of the study area while Group B soil has a similar share with almost 32% of the study area.

Curve number (CN)^117^ is an indicator of the land's capacity to absorb water into the soil, and the higher its value the lower the infiltration rate. For the Curvature Subcarpathian region, the soils having low infiltration rates (CN > 70) covers almost 60% of the area, a situation that favors runoff and surface erosion.

Rainfall Erosivity, known as R-factor^88^, is another indicator of soil exposure to erosion caused by rainfall drops. This indicator was taken from the “European Soil Data Center—ESDAC”, and represents the multi-annual erosion capacity of rain. For the study region, over 70% of the land has an average rainfall erosivity of over 800 (Mj mm/ha h yr), which can be considered as medium to high value compared with values across the country. Soil Texture^115^ in the study region is dominated by the Clay Loam class (> 81%), which corresponds to soils with low permeability, favoring runoff and erosion.

The SRs density data were derived by considering the number of goats and sheep in each LAU^118^ to the official area of land used as pastures in the cadastral evidence (AGR 101B, 2021). The 2010 share of SRs has been transferred to the 2019 counts at the county level per each LAU, thus, a more recent and accurate density is acquired^119^. This data has been prepared into a raster of the densities per each LAU, and it shows high variability in space making it a good explanatory variable. It is spatially non-homogeneous and exhibits a bell curve distribution.

Pasture surfaces data were obtained from the National Institute of Statistics of Romania by considering the land fund corresponding to “pastures' of each Local Administrative Unit at level 2 (LAUs) within the Subcarpathians region^120^. Curvature Subcarpathians region shows a high national average stocking density of SRs (sheep and goats) of about 4.7 units per ha, with uneven territorial distribution (SD = 9.26) (see Supplementary 1).

The spatial distribution of the factors described above (Fig. 4) highlights, on the one hand, the importance of their selection in applying the final index, and anticipates that grazing activity is one of the factors that control erosion activation in the Curvature Subcarpathians region.

### GSLDI application

**Figure 5:**
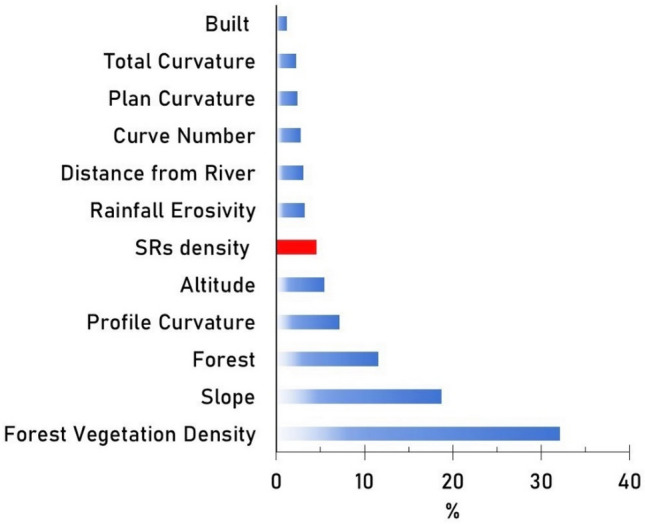
Conditional factors and their relative influence (%) / share/rank (%) to land degradation in the Curvature Subcarpathians region under GBM.

The relative influence of the predictors as identified by GBM is presented in Fig. 5. It indicates that grazing of SRs plays a low role in the exposure to land degradation process, being the sixth important parameter among the 17 geographical variables, and contributes to only 4.55% of the whole exposure in the Curvature Subcarpathians region. The ranking of the relative influence shows the highest values for forest (43.7%) and terrain variables (e.g., slope 18.7%; profile curvature 7.19%) in the Curvature Subcarpathians region (Fig. 5).

**Figure 6:**
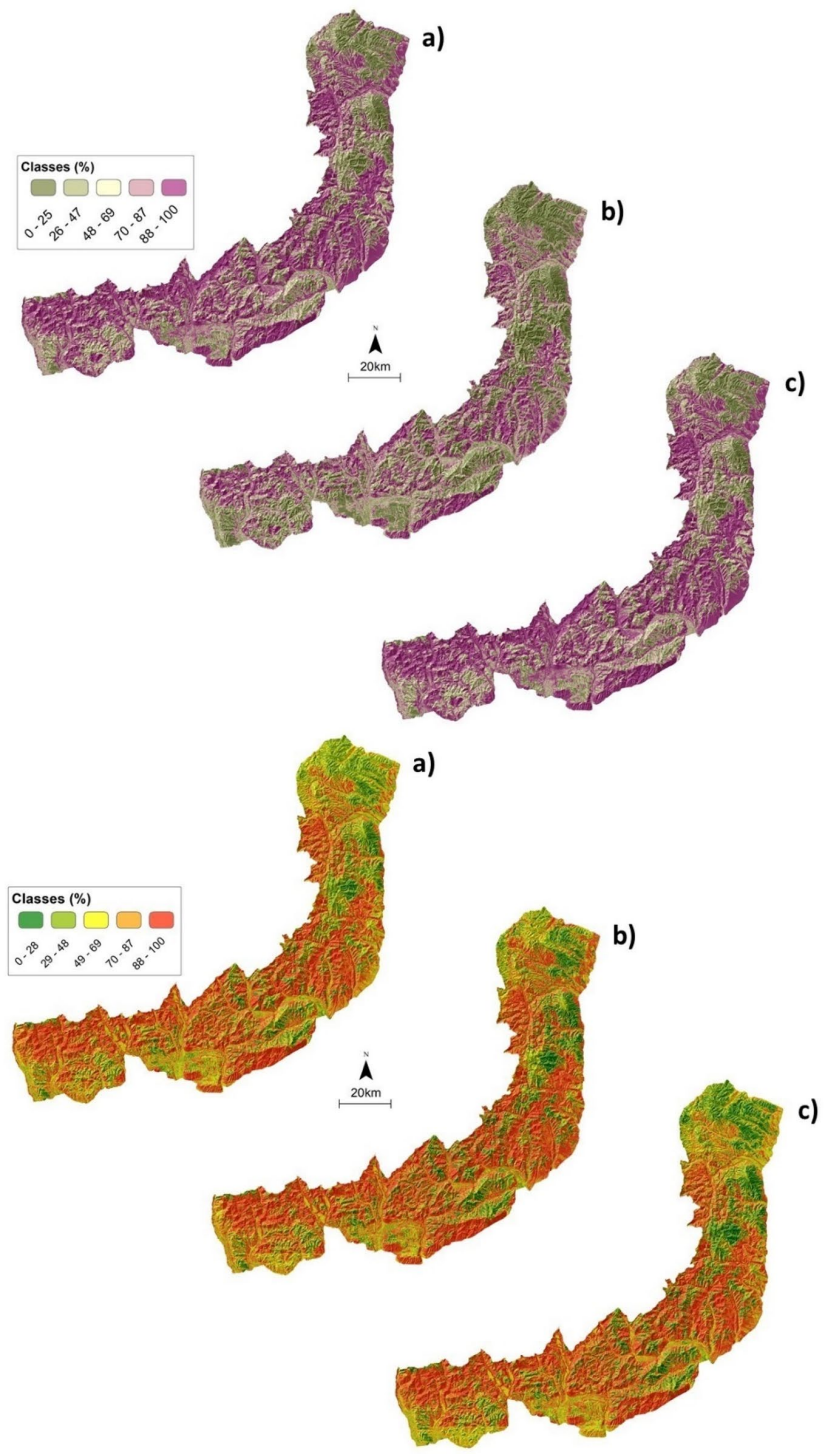
GSLDI probabilistic maps (0–100%) in CS region with Random Forest (top 3 images) and Gradient Boosted Machine (bottom 3 images) under *initial* (**a**), zero (**b**), and 100% overstocking (**c**) scenarios. The map was generated by N. Ciobotaru using the QGIS version 3.20 (https://www.qgis.org).

In order to assess the grazing impact of SRs and build the GSLDI over the study region, we work with two extreme scenarios of absence (0%) and overstocking of 100% (Fig. 6). The absence of grazing pressure signals a decrease of erosion likelihood exposure in the GBM model, while the RF model signals an increase in exposure. An increase of 100% of the grazing pressure indicates an increase in erosion likelihood exposure in both models, suggesting that grazing activity is one of the factors that control erosion exposure in the Curvature Subcarpathians region (Fig. 6).

**Figure 7:**
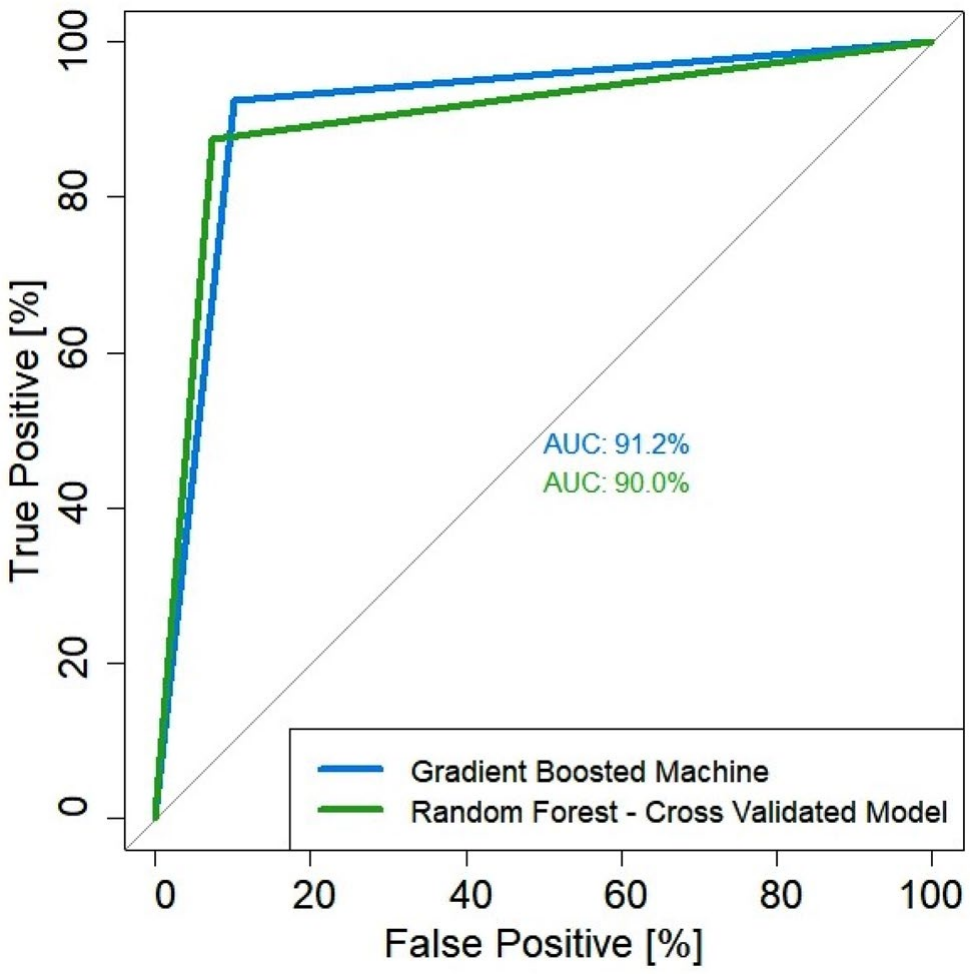
ROC curves with associated AUC values computed from Gradient Boosted Machine and Random Forest.

*Model training* with RF and GBM algorithms performed very well, having the same accuracy of over 90%, and suitable for the mapping of land degradation in the Curvature Subcarpathian region. The ROC curve given in Fig. 7 was generated from the test dataset, containing 20% (830 points) of the points used to indicate the presence or absence of the erosion processes. This set of points has not been used in the training of the models and were randomly selected. Model prediction performance for RF and GBM algorithms was accurate, with an area under the curve (AUC) covering more than 90% of the ROC graph for both models. Therefore, the performance of the models was good enough to be used for prediction the of land degradation exposures in the Curvature Subcarpathian region (Table 2, Fig. 7).

**Table 2:** Model efficiency (%) according to multiple efficiency criteria.

Parameter	Model	
	RF	GBM
Accuracy	0.91	0.94
Sensitivity	0.94	0.93
Specificity	0.88	0.94
Balanced accuracy	0.90	0.94
AUC	0.90	0.91
ROC	0.96	0.98

*RF* random forest, *GBM* gradient boosted machine, *AUC* area under the curve, *ROC* receiver operator characteristic.

## Discussion

The potential value of GSLDI is the ability to connect environmental and human factors. It can be considered as a predictive index encompassing derivatives from the DEM, lithology, vegetation features, and small ruminants’ stock, all being dependent on the soil erosion rates.

The modeling framework involves sampling of erosion and no-erosion points (4187 sampling points), assigning the physical and geographical characteristics (17 attributes) to each sampling point, standardizing the dataset (55 final attributes), and using them in the training of ML models of RF and GBM. The GSLDI indicates that SRs grazing plays an important role, but is not the major factor in the exposure to erosion process, contributing to only 4.6% of the whole exposure to land degradation, according to GMB model parameter rank classification. However, this low percentage agrees with the findings of Nicu^70^ who reported, for a gully assessment in the northeastern part of Romania, that overgrazing does not considerably change the erosion rates. Another explanation of land degradation and the high value of erosion rates could be the lithology and neo-tectonic movements (uplift rates of 3–4 m/year)^121^.

The results have some uncertainty stemming from (1) the low-quality resolution of the biophysical predictors and (2) the required field monitoring for evidence in agricultural catchments for quantitative evidence of erosion rates. Now a critical question here is why the results are different and which one is the truth?

We found for the areas covered by pastures, natural grasslands, or other grasslands (*Sclerophyllous* vegetation, sparsely vegetated areas), the scenario with the absence of grazing pressure signals a small decrease of erosion likelihood exposure with the GBM model, while the RF model signals an increase in exposure (Table 3). This may indicate that the model sensitivity to this factor is low and not enough to observe the impact of the grazing pressure for the RF model. An overstocking of 100% of the grazing pressure indicates a small increase in erosion likelihood exposure for the GBM model, which may indicate again the grazing activity does not play a major role in the erosion exposure in the Curvature Subcarpathians area (Table 3).

**Table 3:** GSLDI mean values (%) for pastures and grassland areas.

Model	Land use/land cover (sq km)		
	Pastures (1229)	Natural grasslands vegetation (163)	Other grasslands (1.74)
RF—0 pressure	0.92	0.94	0.89
RF—base scenario	0.92	0.94	0.9
RF—2 × pressure scenario	0.91	0.93	0.9
GBM—0 pressure	0.87	0.89	0.86
GBM—base scenario	0.94	0.95	0.93
GBM—2 × pressure scenario	0.95	0.96	0.93

The forest plays a crucial role in land degradation through erosion. More precisely, the forest vegetation density has a relative influence share of approximately 33% in explaining the susceptibility to land degradation. Approximately 11% of the results is explained by the simple presence of forest, without regard to any other particular features. This is a very important result confirming that it is not necessarily the presence of the forest that is important, but the presence of tall vegetation with high density that counts. This might explain why authors such as Broeckx et al.^79^ show a very weak correlation with sediment yield in the agricultural catchment with strongly contrasting land uses in their work about sheet and rill erosion rates in Romania. Therefore, for future studies on erosion susceptibility or land degradation we also recommend the use of forest vegetation density instead of simply forest. In the long term, a more adapted classification of grazing range/scale would be necessary to avoid the risk of overestimating potential land degradation areas.

**Figure 8:**
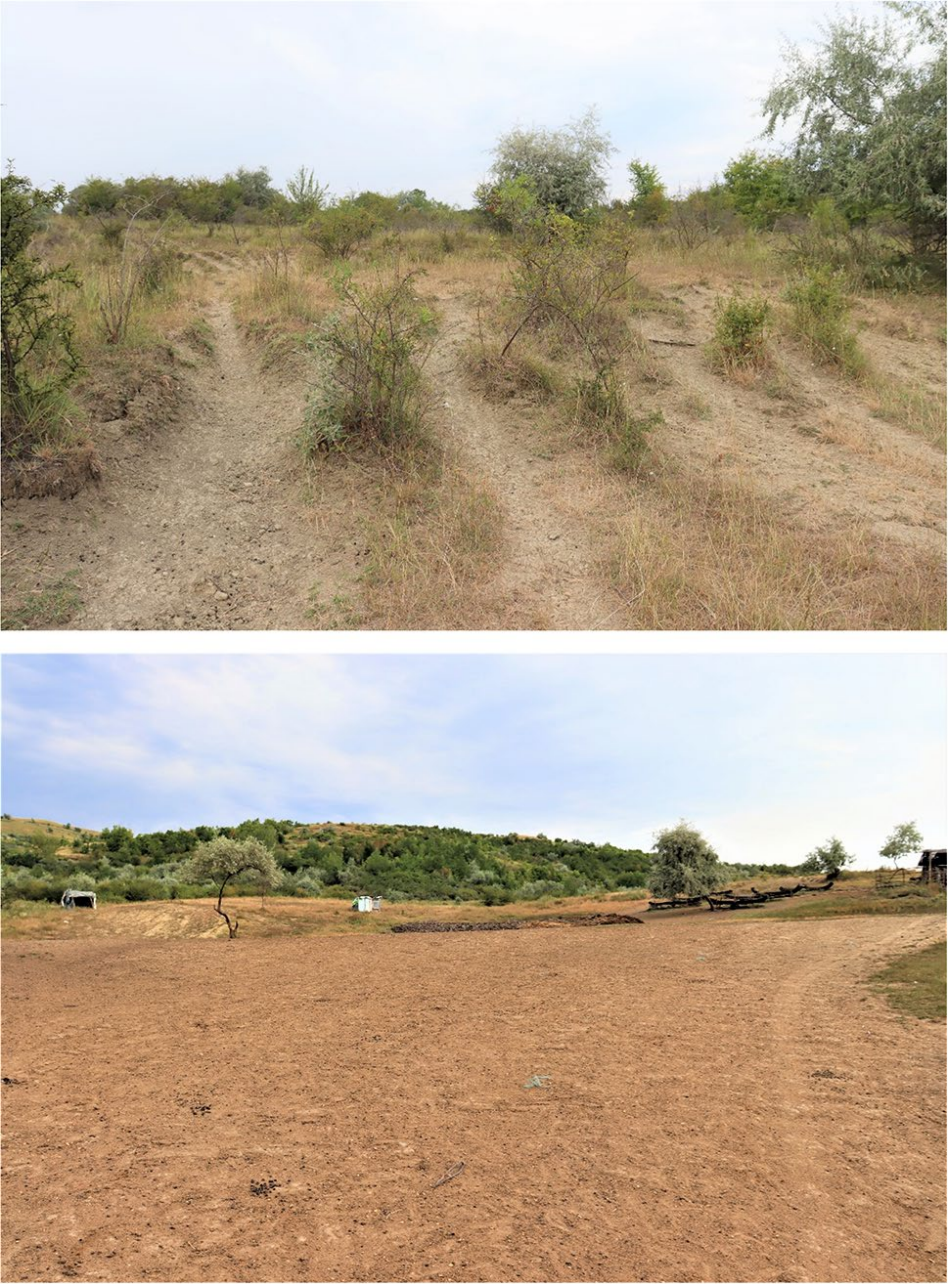
Sheet and linear grazing impact of SRs photos—near paddock (up/left) and trails (down/right) in the Curvature Subcarpathians (Romania); photograph taken by Gabriel MINEA on August 18th, 2021.

We did not exclude or minimize the role of grazing impact of SRs, especially at the local scale (Fig. 8), but over time it will result in complex interactions depending on the environmental factors. However, a quantitative assessment of grazing impact of SRs needs observation and empirical survey (e.g., sediment traps and chemical fingerprinting**)** to understand the hillslope process and the extent of rates of erosion^40,62,122^.

The GSLDI enters the category of GIS tools based on artificial intelligence employed to solve complex issues. Therefore, it suits the analysis of land degradation processes such as soil erosion that integrates numerous factors^123,124^. When compared to similar models focused on land degradation, the GSLDI integrates a pastoral activity (i.e., small ruminants grazing) that is the most common farming practice in certain parts of the world. Therefore, GSLDI is recommended for its use and application for common land management issues.

We encourage managers to use the GSLDI tool for better planning at the local administrative unit scale. As an example, simulations could be conducted by changing the land cover land use or stock/densities of small ruminants in order to identify solutions to mitigate erosion adapted to the morphological features of the studied area. The GSLDI could be a tool employed to find small solutions to a bigger problem, which corresponds to the current non-invasive trend in environmental management and land-use policies.

## Conclusions

In this work, we developed a model, the Grazing Susceptibility to Land Degradation Index, to assess land degradation susceptibility in areas affected by small ruminants grazing. The GSLDI integrates into a GIS environment several factors such as derivatives of topography, lithology, soil characteristics, vegetation features, rainfall erosivity, as well as stock densities of small ruminants. All these variables were associated with sampling points with or without erosion process. The GSLDI was applied in a hilly region that experiences the highest erosion rates in Romania. We found that small ruminants grazing contributes only 4.6% to the erosion of the whole studied area. Overall, we consider that a better knowledge of the relationship between SRs and land use can be beneficial to society and the environment (e.g., soil conservation, hazard management, subsidy, mitigation measures), and the GSLDI can be used efficiently to assess land degradation concerning small ruminant grazing in other land degraded regions.

## Supplementary Information

Supplementary Information.

## Data Availability

Supplementary Material S1.
